# Global surgery research: defend or denounce? Focus on the ethics of research in low- and middle-income countries

**DOI:** 10.1093/bjs/znaf204

**Published:** 2025-12-10

**Authors:** Jenny Edge, Ines Buccimazza

**Affiliations:** Department of Surgery, Faculty of Health Sciences, University of the Witwatersrand, Johannesburg, South Africa; Department of Surgery, Nelson R. Mandela School of Medicine, College of Health Sciences, University of KwaZulu-Natal, Durban, South Africa

## Introduction

Global surgery evolved in response to the perceived neglect of surgical care in global health, particularly in resource-constrained settings. The call to arms was sounded in 2008 by Paul Farmer and Jim Kim who described surgery as ‘the neglected stepchild of global public health’^1^. This clarion call was embraced by the *Lancet* Commission on Global Surgery, which promoted global surgery as a field with the primary aim of empowering underserviced and marginalized populations to improve health outcomes through research, policy development, and advocacy^2^.

However, in places, the noble aspiration of global surgery has been usurped. Over the decades, there have been concerns about low- and middle-income countries (LMICs) becoming ‘victims of scientific colonialism’^3^. This topic is further addressed in the article by Qin *et al*. in this issue^4^.

This paper will concentrate on the ethical and professional issues relevant to global surgery research.

Good-quality, ethical surgical research is essential for the planning for provision of effective healthcare to the estimated 2 billion people worldwide who do not have access to surgery at present. It also leads to surgical advances and improved patient outcomes.

The burden of surgical disease seen in LMICs is different from that in high-income countries (HICs). Infectious diseases, such as human immunodeficiency virus (HIV) infection and tuberculosis (TB), are more common in LMICs^5^. However, the incidence of non-communicable diseases is increasing to account for more of the healthcare burden. In emerging countries, when compared with HICs, there are fewer resources available and access to healthcare is problematic so that patients tend to present with diseases at a more advanced stage^2,6^. Appropriate surgical research assists with resource planning, tailored disease management, and development of innovative ideas.

In this article, some ethical components of global surgical research are discussed, highlighting where these are lacking and how they can be mitigated.

## Research quality

There are many excellent examples of research from LMICs. Some of these, although conducted in LMICs, have resulted in practice changes across the whole world. Notable examples include studies on the use of inexpensive mosquito nets for hernia repairs. These have been shown to be non-inferior to the more expensive commercial meshes. Despite widespread uptake of inexpensive mosquito nets in LMICs, most hernia surgery in HICs is performed with expensive commercial meshes. Therefore, the question remains as to why the cheaper mosquito nets are not being used in HICs as well^7,8^.

A study done in Cape Town showed that HIV-positive patients can be kidney donors for HIV-positive recipients requiring transplantation. This has been accepted as standard of care. The research was conducted due to the high number of patients with HIV in South Africa, but has been of benefit to the whole surgical transplant community^9^.

## Research skills and capacity

Many reasons have been advanced for why African research capacity has not paralleled that of HICs. One reason is that few clinicians in Africa are trained in research^10^. In 2009, a report by the Academy of Science of South Africa (ASSAf) identified a dearth of clinically trained research scientists who could lead research to address challenges in African healthcare and train future clinical researchers^11^. Thus, collaborating with HICs has the potential to fill this deficit, by bringing the required skills, funding, and resources to Africa. However, there is potential for a power imbalance in these relationships. HIC academics, who work for universities that use research output to measure success, have been accused of extractive research—exploiting local resources and reaping the benefits of the resulting publications^12^.

Conducting surgical research in Africa presents unique ethical challenges, particularly given the lack of guidelines for tackling these challenges and other considerations facing global surgery collaborations^13^.

LMIC partners need to be empowered with research skills. A recently published web-based survey among global surgery practitioners highlighted the inequitable access to academic networks and education^14^. HIC institutions can transfer research skills by facilitating access to distance learning resources, protocol development, statistical expertise, database development, and management^15^.

## Research agenda and priorities

For research in LMICs to be conducted with equivalent ethical standards to that in HICs, it is essential that both researchers and patients or other subjects are involved in all stages of project development, encompassing the six commonly used stages of research projects: conception, ethical approval, data collection, analysis, report writing, and article dissemination^16^.

Conception and the research agenda should arise from within the community where the research is being performed. Any study should be of relevance to the community and result in tangible lasting benefit to the community. The lead must come from local researchers who set the research agenda, provide guidance to international researchers, ensure that research collaborations align with LMIC national priorities, prevent research duplication, and ensure that each project demonstrates mutual and equitable benefit. For example, the Ubuntu Clinic, which treats HIV and TB patients in Khayelitsha township, South Africa, is a principal example of how local research committees can inform and guide appropriate research projects^17^.

The needs of a community are identified by listening, which is an active process and one that many health practitioners are poor at doing. Nuances of communication cannot be gauged online and it cannot be assumed that a problem in one area is like another. Ideally, appreciation of the facilities involved and the patient demographics should occur before conception of a research project. For example, during a recent project in Southern Zimbabwe, the hospital management was asked about the main means of communication and they replied ‘donkeys’—which are in fact the main means of transportation. This information would not have been elicited through online chats.

Many global surgery (and similar) departments in HIC universities initiate a growing number of projects, for which they then wish to recruit LMIC surgical collaborators. It is impossible to know how many of the projects currently undertaken in LMICs are initiated by researchers from HICs. While their input can be very beneficial in terms of time, resources, and research expertise, it can lead to inappropriate, non-sustainable studies and poor research. This is referred to as the ‘helicopter’ effect. A recent editorial in *Nature Geoscience* highlights the unequal nature between well-resourced institutions and LMICs, noting that this can result in poor research that is ethically compromised; in the same article, they also encourage lead investigators to include all collaborators as authors^18^. The number of HIC researchers wanting to work in African settings needs to be regulated and limited to those with a genuine desire to collaborate, empower, address locally identified priorities, and treat local counterparts as equals.

## Ethical issues and approval processes

Poor research and research misconduct are not solely surgical or LMIC problems. Historically, it is known that many brilliant people have also done research that could be seen as flawed in quality and/or ethics. In contemporary times, Peter Wilmshurst, a vocal critic of the evidence produced as part of ‘evidence-based medicine’, has published numerous articles on the quality of research on which clinical decisions are based^19,20^. Journal editors have expressed concerns about research quality and integrity, and mechanisms are in place to try to prevent unethical studies being undertaken (via requirements for pre-study permits and via regulations on scientific publishing)^12^.

The foundation stone of ethical considerations is summed up in the Declaration of Helsinki: ‘While the primary purpose of medical research is to generate new knowledge, this goal can never take precedence over the rights and interests of individual research subjects.’; therefore, ethics review boards are needed to provide additional oversight and to ensure that all studies comply with international ethical standards, including protection against exploitation of vulnerable local populations^21^.

Most HICs have robust systems of local or national ethics committees that oversee clinical research projects and the introduction of new therapies and techniques in clinical practice. However, in LMICs, such committees are not universally available, have a varying scope of oversight, and may have a limited range of expertise and resources. The lack of research ethics committees in LMICs has been cited as a reason for the lack of research emanating from LMICs^22^, but a survey conducted by Ermich *et al*.^23^ found that there are 176 research ethics committees in 36 countries in Africa and that the majority are in South Africa. In 2025, in South Africa, at the time of writing, there are 48 ethics committees registered with the National Health Research Ethics Council^24^. Ermich *et al*.^23^ found that the main self-identified functions of research ethics committees are to assess research projects for relevance, feasibility, and consent; most of them (97%) are institutional and meet a variable number of times per year (1–48 times).

The process of getting research ethics committee approval for a project can be very time-consuming, cumbersome, and expensive. Clarke^25^ assessed the process of getting ethics approval for a Master’s study in South Africa (which relatively speaking has many research ethics committees) and found it to take approximately 4 months.

The purpose of ethics approval is not only to ensure that the research adheres to the Declaration of Helsinki but to also give an idea as to whether the research is needed in that community and will result in benefit to that community. The training of academics locally in the principles of ethics and the establishment of ethics committees have been largely ignored by donor countries. This needs to be redressed. Recently, the University of KwaZulu-Natal has started a training programme, the South African Research Ethics Training Initiative (SARETI), to address the lack of training^26^.

A scoping study, looking at published literature about the ethical considerations of research carried out in LMICs, noted that 80% of publications were written exclusively by authors from HICs, with only 7% from authors exclusively based in LMICs; furthermore, the majority of these articles were opinion pieces or editorials^13^.

## Data collection

Modern data collection is largely dependent upon an adequate supply of electricity and Internet provision. With the development of off-the-grid electrical supplies such as solar and the Internet being more universally available, the capacity to collect data has increased. However, it takes time to build up a database. Retrospective data collection is notoriously unreliable and in many instances is dependent upon paper notes with their attendant problems. It is essential to understand the record keeping system at a particular facility before any research is started.

There is also an ethical conundrum regarding the ethics of employing data collectors in LMICs. The salary requirements for such workers are significantly lower than for researchers in HICs, but offshoring large volumes of work may not be the ethical way to obtain data. The quandary is that a research project financed by an HIC institution can provide employment and development opportunities for local workers and is often popular with local individuals.

## Data analysis

Analysis of data requires statistical skills and tends to be done in universities or the research and development departments of commercial organizations. For the most part, this is reasonable and is a beneficial example of HICs supporting LMICs. However, capacity building of research skills within LMIC universities and similar institutions should be an important prerequisite of funding for global surgery projects.

## Reporting

Although there are many well-established and long-standing partnerships between surgeons in LMICs and HICs, the authorship of resulting papers is disproportionately in favour of the HIC collaborators. Over the past decade, various authors have reported that <15% of all global surgical studies are published by LMICs, including those in Africa^2,10^.

A systematic analysis of authorship of global surgery papers emanating from LMICs showed that 21% of all studies had LMIC first and last authors and 59% had HIC first and last authors^27^. Similarly, a review of 786 779 global health publications showed that 86% included at least one LMIC-affiliated author and a modest yearly increase in first and last authors affiliated with LMICs^28^.

In 2023, Kebede *et al*.^29^ performed a systematic mapping review of research in global surgery, screening over 117 000 articles. They included over 2000 articles in their review and reported that authors from six countries (the USA, India, Brazil, China, South Africa, and the UK) accounted for over 50% of first authors. Disproportionately, 41.6% of first authors and 40.7% of last authors were from HICs. Furthermore, 45.6% of articles had no author from the country where the study took place.

Publications need to be collaborative and include principal investigators from HICs and African partner institutions who are involved in all aspects of each individual project. Token authorship should be strongly discouraged and studies quantifying authorship equity are needed.

## Article dissemination

Research is expensive in terms of the cost of the research and the time taken, but publishing costs and then the cost of accessing research publications can be prohibitive. The late Robert Maxwell famously described scientific publishing as a ‘perpetual financing machine’. In 1946, he was hired by Butterworths to help expedite the publication of British scientific papers. After they abandoned the plan 5 years later, he bought controlling shares in Butterworths and Springer. He amassed a fortune from publishing scientific work done and funded by others, written (without payment) by scientists, and then purchased by academic institutions in which the work had taken place^30^.

The estimated turnover generated by the scientific publishing industry is €16 billion (more than the movie industry)^28^. Unlike newspapers, which have a profit margin of 10–15%, the publishing houses have a profit margin of 40%^31^. This is not surprising as journals do not pay for the research, the writing thereof, or the peer review process.

In recent years, open access publication has increased access to scientific research publications, but has increased the overall cost of publishing, which limits the ability of small research teams and independent researchers to disseminate their research findings widely. Kilgallon *et al*.^32^ have estimated that the median cost for publishing an article in an open access journal is nearly €4000.

The real cost of paying for publishing a scientific article for researchers from LMICs is highlighted very eloquently in a letter published in *Nature* in which the cost of publishing an article, without including all of the hidden costs such as the costs to the patient and the cost to the institution, was equivalent to 6 months’ pay for an academic^33^.

## Patient participation and protection

Although participation in research projects may be of clinical benefit to patients, there are costs to subjects in terms of time and transport. In LMICs, where not working is equivalent to not earning, taking time to make extra visits to a hospital or clinic can have an impact on the patient’s and their family’s finances. In South Africa and many other LMICs, transport is the greatest barrier to access to care. In many poor communities, the main mode of transport may in fact be a donkey. Costing the donkey trip to the health facility is problematic. For many patients this means they also need to stay overnight near the health facility to make the long journey back the following day. Appropriate compensation should be paramount to any grant proposal, but reimbursement for participants, beyond a nominal fee, is not generally applied.

In the UK and many other HICs, patients have rights over their tissues or data, and donations are made on an altruistic basis after a detailed consent process. This is not always replicated in LMICs, where biobanks are increasing in popularity. Consent for donation is often cursory. Furthermore, these biobanks may be owned and used by commercial organizations for financial gain. Ethical issues include consent from patients, equity issues about data sharing and processes, investment in human resources and collaborative relationships, and capacity building approaches^34^. LMICs must take the lead and ensure that the biobanking of tissues from their populations will result in research that benefits their populations. At present, many surgical patients cannot afford basic immunohistochemistry—for example, in South Africa, the cost of histopathology and immunohistochemistry for a breast cancer biopsy is equivalent to about 60 months of tamoxifen (calculation based on the cost of tamoxifen to patients in the province of Gauteng and the cost of histopathology and immunohistochemistry in February 2025).

Occasionally, research on human tissues leads to discoveries with significant and long-lasting commercial reward. In 1951, Henrietta Lacks had her cervical cancer biopsied and, unbeknown to her, the biopsy gave rise to the immortal HeLa cell line. This was used for research and commercially, both without her consent, and is discussed further in Skloot’s book *The Immortal Life of Henrietta Lacks*^35^.

The ethics of both specimen collection and usage during tissue biobanking have remained controversial.

In 2018, the Global Forum on Bioethics in Research was held and discussed the ethics of biobanking tissue and sharing patients’ data^34^. Ideally, biobanking tissue and sharing patients’ data should be mutually beneficial to researchers and the population whose tissue is being biobanked or whose data is being shared, and should be promoted to increase surgical research. However, the individual’s rights need to be observed. At the forum, key elements were identified. These included that the process should be transparent, involve the communities whose data or tissues are being used, be non-exploitative, and have no conflict of interest. Although there is some legislation to protect the rights of individuals (for example the Protection of Personal Information Act (POPIA), which was passed in 2020 in South Africa), this area of research is increasing and remains largely ungoverned. Surgeons are often involved in obtaining specimens for biobanking and are in a position of trust with patients at a vulnerable time. It is essential that patient autonomy is respected and that informed consent for retention of tissues or data for research is obtained in an ethical manner and not just as an addendum to consent for treatment. This is particularly pertinent when the researcher does not speak the patient’s first language and when the research is unlikely to be of direct benefit to the patient or their community.

## Summary and conclusions

Despite global surgery being embraced as a subspecialty in many academic institutions, the control of surgical research in LMICs remains with HICs. Before this paradigm can be shifted, expertise in research techniques, grant writing, and ethics committees must be embedded in LMICs. In turn, with overseas funding becoming scarcer, innovative means of generating sustainable private public partnerships should be explored.

In 2005, Volmink and Dare^36^ wrote ‘Research with, rather than in or about, Africa is the goal.’. It is a poor reflection that, 20 years later, that goal is no nearer to being reached.

## References

[1] Farmer PE, Kim JY. Surgery and global health: a view from beyond the OR. World J Surg 2008;32:533–536 18311574 10.1007/s00268-008-9525-9 PMC2267857

[2] Meara JG, Leather AJM, Hagander L, Alkire BC, Alonso N, Ameh EA et al Global surgery 2030: evidence and solutions for achieving health, welfare, and economic development. Lancet 2015;386:569–624 25924834 10.1016/S0140-6736(15)60160-X

[3] Trostle J . Research capacity building in international health: definitions, evaluations and strategies for success. Soc Sci Med 1992;35:1321–1324 1462171 10.1016/0277-9536(92)90035-o

[4] Qin R, Jumbam DT, Roy N. Neocolonialism: should it concern surgeons? BJS 2025;112. 10.1093/bjs/znaf203 41369654

[5] Joint United Nations Programme on HIV/AIDS . *2024 Epidemiological Estimates*. https://www.google.com/search?q=UNAIDS+2024+epidemiological+estimates.&oq=UNAIDS+2024+epidemiological+estimates.&gs_lcrp=EgZjaHJvbWUyBggAEEUYOTIHCAEQIRiPAjIHCAIQIRiPAtIBCDEzMTlqMGo3qAIIsAIB&sourceid=chrome&ie=UTF-8 (accessed 13 January 2025)

[6] Alkire BC, Raykar NP, Shrime MG, Weiser TG, Bickler SW, Rose JA et al Global access to surgical care: a modelling study. Lancet Glob Health 2015;3:e316–e323 25926087 10.1016/S2214-109X(15)70115-4 PMC4820251

[7] Freudenberg S, Sano D, Ouangré E, Weiss C, Wilhelm TJ. Commercial mesh versus nylon mosquito net for hernia repair. A randomized double-blind study in Burkina Faso. World J Surg 2006;30:1784–1789 16983472 10.1007/s00268-006-0108-3

[8] Skopec M, Grillo A, Kureshi A, Bhatti Y, Harris M. Double standards in healthcare innovations: the case of mosquito net mesh for hernia repair. BMJ Innov 2021;7:482–490

[9] Muller E, Barday Z. HIV-positive kidney donor selection for HIV-positive transplant recipients. J Am Soc Nephrol 2018;29:1090–1095 29330339 10.1681/ASN.2017080853 PMC5875954

[10] Wireko AA, Ohenewaa Tenkorang P, Tope Adebusoye F, Mehta A, Cheng Ng J, Yaa Asieduwaa O et al The state of African surgical research capacity: highlighting the current efforts, challenges, and recommendations—Editorial. Int J Surg 2023;109:91–93 36799813 10.1097/JS9.0000000000000216 PMC10389421

[11] Academy of Science of South Africa . *A Study on Clinical Research and Related Training in South Africa*. https://www.assaf.org.za/files/2009/09/ASSAf-Clnical-Report-Summary.pdf (accessed 5 May 2025)

[12] Committee on Publication Ethics . *2008 Code of Conduct*. https://publicationethics.org/files/2008 Code of Conduct.pdf (accessed 6 May 2025)

[13] Grant CL, Robinson T, Al Hinai A, Mack C, Guilfoyle R, Saleh A. Ethical considerations in global surgery: a scoping review. BMJ Glob Health 2020;5:e002319 10.1136/bmjgh-2020-002319 PMC7204923 32399258

[14] Seyi-Olajide JO, Brindle M, Faboya O, Sleemi A, Williams O, Ameh EA. Is neocolonialism existing in global surgery practice? An analysis of a web-based survey amongst global surgery practitioners. J Glob Health Rep 2024;8:e2024016

[15] Chu KM, Jayaraman S, Kyamanywa P, Ntakiyiruta G. Building research capacity in Africa: equity and global health collaborations. PLOS Med 2014;11:e1001612 24618823 10.1371/journal.pmed.1001612 PMC3949667

[16] Ajuwon AJ, Kass N. Outcome of a research ethics training workshop among clinicians and scientists in a Nigerian university. BMC Med Ethics 2008;9:1 18218097 10.1186/1472-6939-9-1 PMC2246144

[17] Médecins Sans Frontières and the Infectious Disease Epidemiology Unit, School of Public Health and Family Medicine, University of Cape Town . *Report on the Integration of TB and HIV Services in Site B Khayelitsha*. https://www.msf.org.za/sites/default/files/Intergration_report__HIV_TB_2005.pdf (accessed 5 May 2025)

[18] Tackling helicopter research. Nat Geosci 2022;15:59735968049

[19] Wilmshurst P . Evidence based medicine: can we trust the evidence? Int J Cardiol 2013;168:636–637 23701933 10.1016/j.ijcard.2013.04.192

[20] Wilmshurst P . Fraud in research. Clin Med 2002;2:159–160 10.7861/clinmedicine.2-2-159 PMC4952380 11991102

[21] World Medical Association (WMA) . *WMA Declaration of Helsinki—Ethical Principles for Medical Research Involving Human Participants*. https://www.wma.net/policies-post/wma-declaration-of-helsinki/ (accessed 12 February 2025) 10.1001/jama.2024.21972 39425955

[22] Hossain I, Hutchinson P, Kawsar K, Kolias A, Santos ALD, Esene IN et al The application of medical ethics in the developing countries—a neurosurgical perspective. Brain Spine 2024;4:103921 39493952 10.1016/j.bas.2024.103921 PMC11530861

[23] Ermich I, Thebaut C, Guerchet M, Boumediene F, Preux PM. Research ethics committees in Africa and Latin America. Med Saf Glob Health 2022;11:156

[24] National Health Research Ethics Council . *Home—National Department of Health*. https://www.health.gov.za/nhrec-home/ (accessed 6 February 2025)

[25] Clarke DL . Auditing the process of ethics approval for Master’s degrees at a South African university. South Afr J Bioeth Law 2014;7:23

[26] University of KwaZulu-Natal . *South African Research Ethics Training Initiative (SARETI)*. https://wp.ukzn.ac.za/site-index/south-african-research-ethics-training-initiative-sareti/ (accessed 5 May 2025)

[27] Ravi K, Bentounsi Z, Tariq A, Brazeal A, Daudu D, Back F et al Systematic analysis of authorship demographics in global surgery. BMJ Glob Health 2021;6:e006672 10.1136/bmjgh-2021-006672 PMC8527109 34666988

[28] Dimitris MC, Gittings M, King NB. How global is global health research? A large-scale analysis of trends in authorship. BMJ Glob Health 2021;6:e003758 10.1136/bmjgh-2020-003758 PMC7843301 33500263

[29] Kebede MA, Tor DSG, Aklilu T, Petros A, Ifeanyichi M, Aderaw E et al Identifying critical gaps in research to advance global surgery by 2030: a systematic mapping review. BMC Health Services Research 2023;23. 10.1186/s12913-023-09973-9 PMC10478287 37667225

[30] Buranyi S . Is the staggeringly profitable business of scientific publishing bad for science? The Guardian 2017

[31] Hagve M. *Pengene Bak Vitenskapelig Publisering*. Tidsskr Den Nor Legeforening. 2020. https://tidsskriftet.no/2020/08/kronikk/pengene-bak-vitenskapelig-publisering (accessed 11 January 2025) 10.4045/tidsskr.20.0118 32815337

[32] Kilgallon JL, Khanna S, Dey T, Smith TR, Ranganathan K. Open(ing) access: top health publication availability to researchers in low- and middle-income countries. Ann Glob Health 2023;89:40 37304940 10.5334/aogh.3904 PMC10253233

[33] Mekonnen A, Downs C, Effiom EO, Razafindratsima O, Stenseth NC, Chapman CA. What costs half a year’s pay for African scholars? Open access. Nature 2021;596:189 10.1038/d41586-021-02173-7 34376836

[34] Bull S, Bhagwandin N. The ethics of data sharing and biobanking in health research. Wellcome Open Res 2020;5:270 33225074 10.12688/wellcomeopenres.16351.1 PMC7670475

[35] Skloot R . The Immortal life of Henrietta Lacks. London, UK: Picador, 2018

[36] Volmink J, Dare L. Addressing inequalities in research capacity in Africa. BMJ 2005;331:705–706 16195259 10.1136/bmj.331.7519.705 PMC1239957

